# Editorial Department

**Published:** 1856-05

**Authors:** 


Dr. Charles Ap A. Bowen, of St. Catherines, C. W., formerly Professor of Anatomy in Geneva Medical College, has accepted the place of Demonstrator of Anatomy in the Medical Department of the University of Buffalo, *vice* Dr. John Boardman resigned. Dr. Boardman leaves the school with the regrets of all concerned.

In the appointment of Dr. Bowen the institution has secured the services of a thorough anatomist, and practiced and successful teacher.

